# Prediction of consumers refill frequency of LPG: A study using explainable machine learning

**DOI:** 10.1016/j.heliyon.2023.e23466

**Published:** 2023-12-18

**Authors:** Shrawan Kumar Trivedi, Abhijit Deb Roy, Praveen Kumar, Debashish Jena, Avik Sinha

**Affiliations:** a Business Analytics and Information Systems Area, Rajiv Gandhi Institute of Petroleum Technology, Amethi, India; b Operations Management, Rajiv Gandhi Institute of Petroleum Technology, Amethi, India; c Management Area, Rajiv Gandhi Institute of Petroleum Technology, Amethi, India; d Operations Management Area, Rajiv Gandhi Institute of Petroleum Technology, Amethi, India; e Management Development Institute Gurgaon, India

**Keywords:** Ujjwala scheme, Welfare economics, Public policy, Machine learning, Artificial intelligence

## Abstract

Launched in 2016, the PMUY Programme of the Government of India aimed to provide 8 crore LPG connections to women in rural households over four years. After acquiring a new connection, some households appeared uninterested in ordering subsequent subsidized LPG refills, impacting programme's sustainability, and targeting strategy. We propose a prediction model using “Explainable Machine Learning” to anticipate the beneficiaries' refill frequency with a view to improving LPG-refills and social targeting. In this paper, we suggest an enhanced stacked SVM (ISS) model for classification, which is contrasted with state-of-art ML models: Random Forest (RF), SVM-RBF, Naive Bayes (NB), and Decision Tree (C5.0). Some of the performance matrices that are used to evaluate the models include accuracy, sensitivity, specificity, Cohen's Kappa statistics, Receiver Operating Characteristic curve (ROC), and area under the curve (AUC). The proposed approach, which was validated with 10-fold cross validation, produced the best overall accuracies for data splits of 50–50, 66–34, and 80–20. The "Explainable AI (XAI)" model has also been used to describe how models and features interact, and to discuss the importance of features and their contributions to prediction. The recommended XAI will aid in efficient “beneficiary targeting” and “policy interventions”.

## 1 Introduction

Historically, LPG was primarily utilized for cooking by individuals with medium and high incomes in India until a few decades ago [1]. As time progressed, LPG became the preferred and more cost-effective cooking fuel among the general population. However, those situated just above and below the poverty line were unable to access LPG due to its prohibitive cost, leaving them with no choice but to rely on other solid fuels such as firewood, kerosene, dried cow dung, and coal etc. Unfortunately, using these alternatives to LPG leads to indoor air pollution, poor health, and deforestation [2] etc.

The Indian Government has implemented a range of social inclusion initiatives, including programs such as "Pradhan Mantri Jan Dhan Yojana" (PMJDY) which promotes financial inclusion for all. Additionally, the "PAHAL or Direct Benefit Transfer for LPG" (DBTL) initiative was introduced to transfer subsidies directly to the bank accounts of beneficiaries by integrating Aadhar number, Bank Account and Mobile number of individuals [3]. The "GiveItUp" campaign also provided citizens with the option to forgo LPG subsidies, while the "Pradhan Mantri Ujjwala Yojana" (PMUY) aimed to provide clean cooking fuel to poor households, particularly in rural areas based on the Socio Economic and Caste Census 2011. The Indian government worked to establish a technological framework alongside these social inclusion programs to ensure that their goal of providing inclusive social schemes for the poor was realized.

The Pradhan Mantri Ujjwala Yojana (PMUY) was introduced in 2016 by the Indian government as a flagship initiative. Thanks to its efficient implementation, the program successfully enrolled 4 crore households in under two years. By 2020–2021, PMUY had expanded to over 8 crore households and over 9 crore customers by 2023. As of July 2023, LPG usage in India has reached 99.8 %, with a total of 9.59 Crores PMUY connections. Since then, both central and state governments have launched various versions of the PMUY scheme [4]. Its primary objectives are to eliminate indoor air pollution, reduce fuel collection for household cooking, curb deforestation, and promote women's empowerment and social inclusion. To meet the significant increase in domestic LPG consumers from 19.88 Crore in 2016 to 31.5 Crore in 2023, the government has taken massive steps through Oil Marketing Companies (OMC) to improve the supply chain infrastructure for LPG distribution. During this period, new LPG distributors were commissioned, increasing the number from 17916 in 2016–25386 in 2023. The number of bottling plants also increased from 188 in 2016 to 208 LPG in 2023.

The sustainability of the PMUY scheme is a concern for the Oil Marketing Companies and the Indian Government due to the low refill consumption by beneficiaries. Sales data analysis showed that PMUY beneficiaries consumed an average of 2.98 refills annually, while non-PMUY customers consumed 6.73 refills in 2018–19. The audit report by Comptroller and Auditor General of India (CAG) in their report published in 2019 had highlighted that 17.61 % of PMUY beneficiaries never returned for the second LPG refill cylinder, while 33.02 % consumed only 1–3 refills after the initial purchase. This trend has persisted, with a slight increase in per-capita consumption in 2019-20 and 2020–21, followed by a dip in 2021-22 and 2022–23. The prohibitive cost of LPG refills is the primary reason that deters PMUY beneficiaries from lifting refills, which they cannot afford. Additionally, inadequate LPG supply chain in rural areas, non-existent home delivery of LPG refill by distributors in rural areas, single bottled connections, easy access to conventional fuel in rural areas, etc., contribute to poor upliftment of LPG refill by PMUY consumers. The low refill upliftment by PMUY beneficiaries jeopardizes the objective of GOI for launching the PMUY scheme and its associated frameworks. The fall in expected consumption of LPG affects the utilization of the augmented infrastructures and has direct implications for the achievement of the Sustainable Development Goals (SDG) objectives on clean and affordable energy for all mandated by the United Nations in India.

In the past, studies have looked at why PMUY beneficiaries are not buying refills, but these studies were limited and did not give a nationwide view. To understand why PMUY beneficiaries are not buying refills across India, we can use the Theory of Customer Churn in the social sector. We believe this type of study has not been done before for the PMUY beneficiaries. To do this analysis, we need to classify PMUY customers into High-frequency and Low-frequency segments. Once we have this classification, the Indian Government and OMCs can create targeted policies to help encourage more refill uptake by the consumers.

This research paper introduces a model called XISS (Explainable Improved Stacked SVM), which aims to classify customers into High-frequency and Low-frequency LPG segments. The model is designed to provide answers to various research questions, as follows.

RQ1 What are the notable features / factors which contribute to the classification of customers as High-frequency and Low-frequency customers?

RQ2 How can Explainable Artificial Intelligence (XAI) assist in understanding the relative importance of each factor?

RQ3 How good is the proposed XISS machine learning predicting model in classifying customers into High-Frequency and Low-Frequency segments?

## 2 Literature review

Studies have shown that energy usage has a significant impact on pollution, health, and the environment. In particular, the use of traditional cooking fuel has been studied for its effects on health due to the increase in household air pollution. Various solutions have been suggested based on studies such as the Global Burden of Disease Study, WHO Indoor Air Pollution and Health Database, The Energy Sector Health Impacts Model (E-SHIP), The HAPIN Study (Household Air Pollution Intervention Network), and The Global Alliance for Clean Cookstoves Research and Publications. The impact of conventional fuels, like biomass (wood, crop residues, and dung) and coal, as cooking fuels varies across regions and countries. These fuels are commonly used for cooking in many parts of the world, particularly in low and middle-income countries. Here are some of the key impacts of conventional cooking fuels: 1. Health Impact: The use of biomass and coal for cooking in poorly ventilated indoor spaces can lead to indoor air pollution, which can cause respiratory diseases, especially among women and children who are exposed to smoke on a daily basis. 2. Environmental Impact: Burning biomass and coal for cooking contributes to deforestation because trees are often cut down for fuel. Furthermore, coal combustion releases greenhouse gases and other pollutants that contribute to air pollution and climate change. 3. Time and Labor Intensive: Collecting firewood or biomass for cooking can be time-consuming for women and girls who are often responsible for this task in many cultures. This can limit their educational and economic opportunities.

In the study published in The Lancet journal [5], has highlighted the impact of household air pollution (HAP) in low- and middle-income countries resulting in a rise in respiratory diseases [6]. in the article published in Global Heart has listed the effect of Household Air Pollution (HAP) due to solid fuel use in cardiovascular diseases (CVD) and respiratory illness. Although respiratory illness is the primary fallout of HAP, the study also indicates that CVD also accounts for the substantial number of mortalities due to HAP across the world. The study reiterates the need for policy formation for the promotion of fuel-efficient low-emission cooking mediums in low-income countries around the world.

Governments worldwide have been promoting the use of LPG as a cleaner cooking fuel through policy interventions, such as India's PMUY, Indonesia's PKH, Ghana's LPG Promotion Policy, Mexico's Programa de Sustitución de Energéticos, Kenya's National LPG Project, Brazil's Bolsa Família, Nepal's Subsidized LPG Program, and Bangladesh's LPG Conversion Program are a few prominent policy interventions.

According to a recent study conducted by Ref. [7] using the Probit Estimation model, implementing clean energy in households yields a range of benefits and long-term effects. These benefits include reducing energy poverty, improving health, increasing household income, and lowering medical expenses. However, the study notes that rural populations are slow to transition to cleaner cooking fuel, particularly the adoption of LPG. In a separate study by Ref. [8], it was found that while LPG is a clean cooking fuel with positive impacts on users, the cost of fuel remains a significant obstacle for rural households in India. A broader policy framework is needed to encourage households to fully embrace clean cooking fuel, and LPG may be an ideal solution as a cleaner cooking fuel source. Additionally [2], highlighted the negative consequences of using traditional cooking fuel on health and the environment in rural areas of India. Other studies by Refs. [[9], [10], [11]] also showed why rural populations continue to use traditional cooking fuel sources, as LPG as a clean cooking fuel source is not affordable for them.

### 2.1 PMUY -- Pradhan Mantri Ujjwala Yojana

The Pradhan Mantri Ujjwala Yojana (PMUY) is a significant initiative of the Government of India that was launched on May 1, 2016, under the leadership of our Prime Minister. The primary aim of the PMUY scheme is to provide access to clean cooking fuel, specifically Liquefied Petroleum Gas (LPG), to women belonging to below-poverty-line (BPL) households across India. The scheme is designed to improve the health and well-being of women and their families by reducing their reliance on traditional and more polluting cooking fuels such as biomass (wood, crop residues), cow dung, and kerosene.

Notable features of the PMUY scheme include the provision of free LPG connections to eligible BPL women, which removes the initial financial barrier to acquiring an LPG connection. This includes a free LPG connection kit that typically consists of a gas cylinder, regulator, pipe, and stove. Beneficiaries of PMUY also receive subsidies on LPG cylinder refills, making them more affordable for continued use. These subsidies are directly provided to beneficiaries' bank accounts through the Direct Benefit Transfer (DBT) system. The scheme aims to expand the reach of LPG distribution networks to rural and remote areas, ensuring that even households in hard-to-reach locations can access clean cooking fuel. PMUY emphasizes the health benefits of using LPG for cooking, as it reduces exposure to indoor air pollution and the associated health risks. The scheme also promotes the use of safe and modern LPG stoves. Furthermore, the scheme is focused on empowering women by making them the primary beneficiaries of the initiative. It recognizes the significant role women play in household cooking and their exposure to health risks associated with traditional fuels. Encouraging the use of LPG, PMUY contributes to reducing deforestation, as it reduces the demand for firewood and other biomass fuels. It also helps mitigate greenhouse gas emissions associated with traditional cooking methods.

The Pradhan Mantri Ujjwala Yojana has been instrumental in promoting clean cooking practices in India, improving the health and quality of life for millions of BPL households, and reducing the environmental impact of traditional cooking fuels. It has been a significant step towards achieving the government's goal of universal access to clean cooking fuel for all thus also achieving the SDG objectives.

A recent study conducted by Ref. [12] explored how Business Model Innovation (BMI) can help bridge the gap in public-sector companies, particularly in the LPG business in India, through supply-side and demand-side strategies and technological innovation via digital initiatives. The research analysed the distinct phases of the LPG business from 1955 to 2013, outlining the transition from LPG subsidy to the dismantling of the subsidy regime, driven by OMC (Oil Marketing Companies) and MOPNG. Another study conducted by Ref. [13] assessed the perception of beneficiaries regarding the benefits of the PMUY schemes, an initiative by the government aimed at improving quality of life, social inclusion, and ecological benefits. The study found that education, LPG refill subsidies, and changes in the use of other fuel sources for cooking were responsible for improving the perception of the scheme.

A recent study examined numerous factors such as social class and gender to suggest that policies promoting greater gender equality and social inclusion could lead to more successful implementation of social benefit programs like PMUY [14]. Another study analysed the use of different fuels across the country, considering sociocultural factors and their effects on health, including the use of LPG. This study also examined the impact of the PMUY scheme and the adoption of LPG as the primary cooking fuel, identifying various methods to improve the sustainable use of LPG in households [15].

A study by Ref. [16] highlighted the benefits of the PMUY scheme, including its affordability and health benefits for rural households. The study also described the implementation procedure of the scheme. Similarly, another study by Ref. [17] examined the impact of cooking fuel choices on the health of urban and rural populations.

A study conducted by Ref. [18] in two states named Raipur in Chhattisgarh and Ranchi in Jharkhand used the TOBIT regression model to analyse survey data and determine the adoption of LPG and the factors that affect it. The study found that the adoption of LPG increased due to PMUY, but continuous usage depended on a range of factors, such as behavioural aspects, education, cost of the cylinder, and supply chain of LPG delivery.

Another study by Ref. [19] used the MILP and a DSS to determine how many distributors are needed to cover the policy objective and increase the adoption and penetration of LPG coverage. The study primarily focused on factors such as the caste, class, and gender of beneficiaries to determine the implementation, effectiveness, and adoption of the PMUY policy, which is based on the Socio-Economic Caste Census SECC (2011) data. The study also found that affordability, availability of LPG, and doorstep delivery by distributors were crucial in improving LPG adoption through the PMUY scheme.

A study by Ref. [16] considered various important aspects, including Economic, Technological, Institutional, and socio-cultural factors, to outline the adoption of cleaner fuel by rural households across India. The study also highlighted the initiatives by the Govt of India in the adoption of cleaner fuel through LPG and various other schemes for the promotion of solar and biogas, as outlined by Ref. [20].

A study in rural household of Rajasthan post PMUY Scheme implementation [21] has shown several benefits to the beneficiaries such as environment friendly cooking, additional time for the family, entertainment & hobby, relieve from time consuming firewood collection process as fuel etc. These benefits of the PMUY scheme have also made women folks socially and economically empowered. Usage of Internet by the rural population in adoption of cleaner cooking fuel and slow transition to cleaner cooking fuel over a period of time was outlined by Ref. [22].

### 2.2 Long term sustainability of the PMUY scheme

In a study on energy consumed by households [23], discussed that the transition pattern from one energy source to another cleaner and efficient energy source primarily depended on the income growth of the individual households.

A study in two districts of Bihar on the beneficiaries of PMUY scheme [24] with its perceived benefits has brought out the direct connection between the education of the beneficiaries and the adaptation and sustainability of the scheme on the long term. Another study of the rural area of Nagpur district of Maharashtra on the PMUY scheme implementation and its sustainability over the longer period to make the rural household move to cleaner fuel for cooking identified various barriers such as affordability, reliability, accountability, and viability for the long-term sustainability of the scheme and outlines steps that need to be taken [25].

Analysis of the PMUY scheme and its applicability [26] was done based on the empirical data arrived at the conclusion that though the scheme was successful as per the initial intended purpose but was lacking when it came to sustained adoption of the benefits over the longer period of time. Despite the upfront benefits of free connection, the beneficiaries were not able to uplift the LPG refill cylinders subsequently. Subsequent studies [27,28] on the issues of sustained upliftment of LPG refill by the poor households shed light on numerous factors primary being the affordability factor, income criteria, home delivery amongst others.

A study was conducted [29] to determine the adoption of cleaner fuel in comparison to biomass which is the most popular cooking fuels in rural households of Maharashtra. It identified that various socio-economic factors such as affordability, availability of refill, education etc. determine the sustainability of the PMUY scheme in long term. Study in tribal regions of Odisha has shown similar trend on PMUY scheme adoption [30] and highlighted the issues in long-term sustenance of the scheme and usage of LPG as cooking fuel on continued basis.

Concerns have been raised about the long-term sustainability of the PMUY scheme by both the Oil Marketing Companies and the Indian Government, as noted by Refs. [31,32] and audit report by Comptroller and Auditor General of India, [33]. Comptroller and Auditor General (CAG) in their report has advised GOI to encourage the use of LPG among the beneficiaries of the Pradhan Mantri Ujjwala Yojana (PMUY). The CAGrecommended that financial support should be provided to these beneficiaries for the purchase of LPG stoves and cylinders, while also educating them about the benefits of LPG use. This would help ensure the sustainability of LPG usage.

Table 1 summarises the related work for this study.

**Table 1:** Related research.

Year	Objective	Technique	Findings	Citations
**2023**	Socio economic survey in a remote village of Gujarat and penetration of PMUY scheme.	Sample Survey and Statistical analysis	Awareness of govt schemes is lacking among the population who are mostly in below poverty line and need financial assistance in elevating their conditions	[34]
**2023**	Study to identify the constraints in adoption of LPG by consumers and distributors	Exploratory Research	The study recommends policy interventions to irradicate the constraints in sustainable adoption by PMUY beneficiaries.	[35]
**2022**	Study of PMUY benefits extended to women empowerment through sustainable use of LPG refills	Empirical Study	The study identifies the issue pertaining to low refill uplift and suggests various steps that could be taken.	[36]
**2022**	Adoption of the PMUY scheme and factors that influence beneficiaries on using LPG as cooking fuel as a primary fuel	Empirical Study	Household income and education were the primary reason/factor the use of LPG as primary cooking fuel by the beneficiaries.	[37]
**2021**	Study the benefits of PMUY scheme and clean energy transition by the beneficiaries based on the perception of the benefits of the PMUY scheme.	Regression Modelling	The study found that the beneficiaries find no direct corelation of the intended benefits such as standard of living and LPG connection from PMUY scheme.	[13]
**2021**	To report exact penetration of LPG across the country and the detailed consumption pattern	Statistical analysis	Provided hard facts of LPG usage and its exact number of beneficiaries in each of the states across the country	[38]
**2021**	Study the benefits of PMUY scheme rolled out from 2016 to 2019 and its effectiveness as per its initial objective of the scheme	Semi-structured interviews and data analysis	The study found out value of time, speed and flexibility, value of saved time as primary benefits of the LPG usage from the PMUY beneficiaries rather than the objective of social inclusion, clean cooking fuel medium etc in the PMUY scheme	[39]
**2020**	Case study to discuss in detailed about the PMUY scheme and its benefits and its achievement till 2020	Sample Survey and Statistical analysis	Generic details of the PMUY scheme and various schemes that lead up to the launch of PMUY scheme.	[4]
**2020**	Study intended to find the adoption of LPG as clean cooking fuel by the PMUY beneficiaries	Empirical Study	Found that the LPG consumption by PMUY beneficiaries was lower than the non-PMUY beneficiaries. Subsidy for the PMUY connection was a success but continued adoption of subsequent refill of LPG cylinder was concern for the BPL households	[26]
**2020**	Analyse the impact of the PMUY scheme on the socio-economic status of women in rural areas	Survey based approach	The study found that there was a huge socio-economic impact of PMUY scheme on the women in rural areas and had achieved a lot in women empowerment.	[21]
**2019**	To analyse the penetration of the PMUY scheme in Nagpur district of Maharashtra & study issues and challenges in adoption of the scheme while switching to clean cooking fuel, LPG	Stratified random sampling technique	The PMUY scheme had achieved its primary objective of providing LPG connections to the poor household but the problem of sustained use of LPG still remained for the beneficiaries	[25]
**2019**	Case study to determine the fuel use pattern and trend in India and the socio-cultural, economical, and behavioural factors that influence the shift to clearer fuel	Decision support system for Pradhan Mantri Ujjwala Yojana	Major finding was that 90 % of the rural population use non-clean fuel for cooking and only 2 % population has adopted clean fuel for cooking from 1993–94 to 2009-10	[15]
**2018**	Analyse integration of LPG as clean cooking fuel in rural household in six states in India	Analysis of survey data	Fuel cost is one of the primary factors in clean Fuel adoption; Dual use of various fuel other than LPG is the generic norm; LPG has greater prospect of adoption in rural India	[40]
**2018**	To explore how PMUY scheme impacted the social inclusion of poor and marginalized section of Indian Society through cleaner cooking fuel adoption using LPG	Empirical Study	Study found that the accessibility to cleaner fuel and affordability is the primary issue for the rural poor in India	[16]
**2018**	Quantitatively analyse the implementation of the PMUY Scheme	MILP Technique	Estimated the number of dealers required to sustain the LPG penetration based on the govt projections and the PMUY scheme	[19]

### 2.3 Research Gap Analysis

**Fig. 1:**
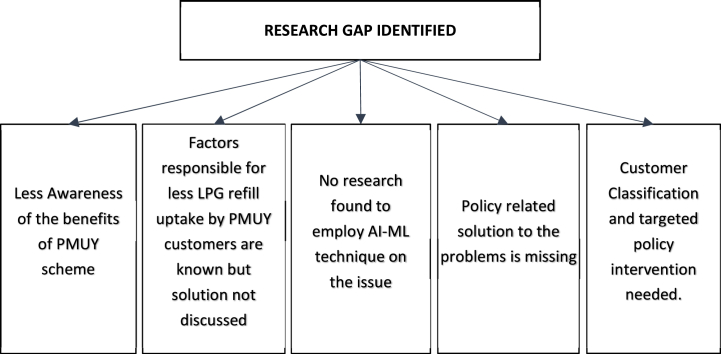
Gap analysis.

Since its launch, the PMUY scheme has been a popular subject of research. Studies have been conducted to analyse its policy matters, benefits, and objectives. Some studies have focused on specific areas to identify the challenges faced by beneficiaries when using LPG sustainably. All studies have emphasized the sustainability of LPG issues and have identified probable reasons such as the prohibitive cost of LPG refill, inadequate supply chain, non-existent home delivery mechanism, and lack of awareness of benefits. However, there has not been a pan-India study using AI/ML to segment beneficiaries into diverse groups based on the theory of customer churn. This research aims to segment beneficiaries and suggest ways to promote sustainable LPG usage among them wherein OMCs and GOI can device policy objectives for the segments thereby enhance the LPG usage amongst the PMUY beneficiaries. Fig. 1 depicts the research gap identified for this research.

## 3 Methodology

**Fig. 2:**
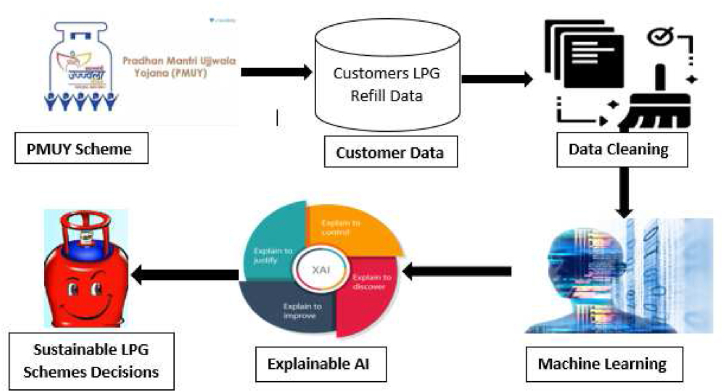
Framework of this study.

Fig. 2 shows the research framework of this research work. From the framework, it can be seen that the PMUY program generates sales and customer profile data. A summary of the PMUY beneficiary information is obtained and put through a data-cleaning process to prepare it for AI/ML modelling. Once the data cleaning process is complete, it is passed through various AI/ML models and their observations recorded. The output from different models is then compared and passed through another phase where XAI (Explainable Artificial Intelligence) analyses the recorded output and provides an interpretation in a human-comprehensible form to aid decision-making.

### 3.1 Data description

This study has used sample data set having demographic, socio-economic and historical information of refill booking by LPG customers. The primary data for this study has been sourced from researcher who sourced the same from OMCs billing platform which caters to the end-to-end process of Customer Enrolment, Refill Booking, Deliveries of LPG refill cylinder, grant of subsidies etc. and all other allied processes of day-to-day LPG Sales business.

The sample dataset selected for study comprises of randomly chosen 8000 PMUY customers from each of the thirty-five states and Union territories of country. This constituted customers from 329 districts, 3020 different towns and villages spread across 1349 different pin-codes. Representation from all eight eligible sub-types of PMUY categories was observed. The dataset was found to comprise of 16 % of urban populace vs 84 % from rural populace. All the features of the data have been shown in Table 2.

**Table 2:** Data description.

S/N	Feature Name	Description
1	DISTRIBUTORCODE	Code of LPG Distributors
2	CONSUMERNUM	Unique code of a customer
3	STATE	State of Union of India
4	PINCODE	Pincode of a place in India
5	URBANRURAL	Urban and Rural Classification
6	RELATIONSHIPSTARTDATE	PMUY connection issue date
7	LASTREFILLDATE	LPG refill last taken date
8	DELIVERYTYPE	How the LPG refill delivery done
9	SUSPENSIONREASON	Reason for customer's connection suspension
10	SCHEMEONBOARDINGSTATUS	LPG Connection status
11	SUBSIDYSTATUS	DBTL status for Subsidy
12	SCHEMESUBTYPE	Sub-type under which the connection was given
13	CONSUMERTYPE	No of LPG cylinders in the connection
14	FREQUENCY	Target Variable computed by the ISS Model

## 4 Machine learning classifiers

A machine learning classifier refers to an algorithm utilized in supervised learning that classifies data into predetermined categories based on patterns derived from a labelled training dataset. It leverages historical data to predict the class labels of new, unseen data points. The classifier learns the relationships between input features, such as email content, image pixels, or text, and class labels like spam/not spam or sentiment. Once trained, it can generalize this knowledge to make predictions on new data. Machine learning classifiers are indispensable in applications that require data classification, such as image recognition, medical diagnosis, and natural language processing. For this study, we have compared the performance of machine learning models namely Random Forest Classifier, Naïve Bayes, C5.0 Decision Tree Classifier, Support Vector Machine (SVM), and Improved Stacked SVM Classifier.

Machine learning, for a segmentation problem, offers improved accuracy, is flexible and adaptable, and saves time and effort. Businesses can use it to segment customers and markets, while image and text segmentation is useful for medical image analysis, object detection, and information extraction. Machine learning algorithms are superior to classical feature extraction methods in terms of accuracy, flexibility, and robustness. Therefore, they should be used to automatically extract informative and predictive features from data to improve segmentation accuracy.

A brief description of few of the machine learning classifiers is as under.

### 4.1 Random forest classifier

The random forest classifier is a widely adopted approach for handling high-dimensional and skewed data due to its exceptional performance [41]. The name of this model originates from the fact that the number of decision trees utilized in the model is selected at random. Given a dataset with D data points and m features, each decision tree is trained by randomly selecting rows and features. In cases where the sampled data has fewer rows or columns than D and m, respectively, the decision tree is still trained in the same manner. The random forest model employs multiple decision trees, each with high variance. However, by aggregating all the decision trees, a majority vote is used to convert high variance to low variance. It is noteworthy that random forest remains an accurate model even when there are only a limited number of data points available.

### 4.2 Naïve Bayes

The Naïve Bayes algorithm is a supervised learning method that employs the Bayes Theorem to address classification problems. As a probabilistic classifier, it predicts outcomes based on an object's likelihood. Naïve Bayes is highly regarded in various domains due to its simplicity. Nevertheless, its fundamental assumption of independence among features is both a strength and a limitation. While this assumption contributes to its robustness, it also results in a loss of accuracy [42] that constrains its applicability in real-world scenarios [43]. Nevertheless, Naïve Bayes remains a desirable choice in situations where large datasets are involved, as it offers faster and less complex performance compared to other machine learning classifiers.

### 4.3 C5.0 Decision Tree Classifier

C5.0 is a robust machine learning system that employs decision tree algorithms and finds extensive practical applications. In comparison to other decision tree-based classifiers, such as C4.5 and ID3, C5.0 boasts numerous advantages, including faster processing speeds, higher precision, and superior memory utilization [44]. As noted by Ref. [45], C5.0 is an extension of C4.5 and ID3. C5.0 excels in handling data that has noise and missing values, and it is adept at addressing overfitting and pruning errors [44]. Furthermore, C5.0 exhibits no concerns with dimensionality in prediction. The outcomes produced by this classifier are readily comprehensible and visually interpretable, thereby obviating the need for advanced mathematical knowledge.

### 4.4 Support vector machine (SVM) classifier

Support vector machine, a supervised machine learning algorithm, can be used for both numerical prediction as well as classification and it can accept both linear and nonlinear data as input [46]. This study uses SVM to classify the intention to use social media for education. SVM has several functional areas such as-identifying handwriting, identifying any object, and identifying the speaker etc. [47] used SVM to identify cancer disease in an individual using gene expression data as input. The most significant advantage of SVM is that its training time is extremely short in comparison to other ML algorithms, while producing high accuracy due to its ability to handle nonlinear data by establishing nonlinear decision boundaries. SVM is less prone to overfitting than other methods. SVM uses optimization by looking for the maximum marginal hyperplane determined by support vectors. The hyperplane mathematical equation is AX*b = 0, where A represents the weight vector, namely A = a1, a2, a3, an, where n represents the number of features, b is a scalar quantity representing a bias in the model, and X represents the feature vector.

### 4.5 Improved Stacked SVM (ISS) classifier

This study proposes a combined classifier that is built by stacking random forest (RF) and support vector machine (SVM) classifiers. The training subset is used to train random forest classifier, resulting in three distinct trained random forest classifiers. The classification decision of the RF classifiers is used as input for the meta-classifier, which is a support vector machine (SVM) that makes the final classification decision [48,49].

### 4.6 Explainable Artificial Intelligence (XAI) or explainable machine learning (XML)

Explainable AI (XAI), also known as Interpretable AI or Explainable Machine Learning (XML), is a burgeoning field of research that seeks to increase the comprehensibility of machine learning models for humans. The opacity of many machine learning models makes them difficult to interpret, necessitating the development of Explainable Artificial Intelligence (XAI) or Explainable Machine Learning (Explainable ML). These techniques, methods, and models within the domain of artificial intelligence and machine learning are intended to enhance transparency and provide insights into the decisions and outputs of AI systems, allowing users to make informed judgments about model outputs and fostering trust.

The primary objective of XAI is to generate explanations for why a particular AI model made a specific prediction or decision. This ability, in turn, creates trust and enables users to assess the model's output. XAI's key characteristics include interpretability, transparency, explanations, model types, trust, accountability, and human-centeredness. XAI models are designed to be interpretable, which means that even non-experts can easily understand and make sense of their internal workings. XAI emphasizes transparency in the decision-making process of AI models, making the process understandable and traceable. XAI generates explanations for AI model predictions or decisions in a variety of formats. This feature enhances trust in AI systems by providing insights into the model's reasoning. It also facilitates accountability by allowing users to determine whether the model's decisions align with ethical, legal, and business requirements. XAI prioritizes human-centred design, focusing on providing explanations that are meaningful and relevant to end-users or domain experts.

To explain the results of machine learning models using XAI, the following approach is recommended: select an appropriate XAI technique based on your model and use case, apply the chosen XAI technique to generate explanations for individual predictions or model behaviour, present the explanations in a user-friendly format, and continuously assess the effectiveness of your explanations and gather feedback from users. By integrating XAI techniques into your machine learning workflows, you can improve the transparency, accountability, and user-friendliness of your models. This is particularly important in applications where understanding the reason behind predictions is critical, such as healthcare, finance, and autonomous vehicles.

**Fig. 3:**
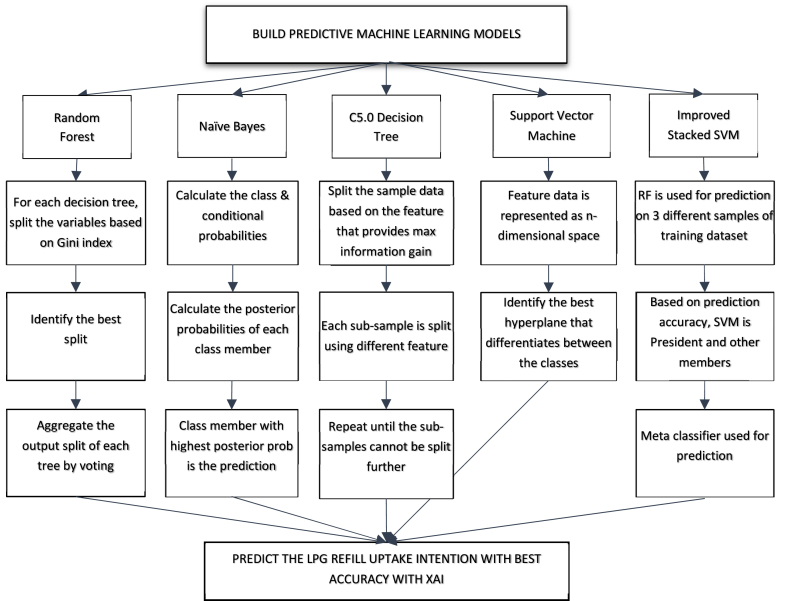
Working of the ML predictive models.

Fig. 3 shows the working of all the models in the study.

### 4.7 Evaluation metrices

In order to analyse and evaluate the output of AI/ML models on a single dataset, we have to take help of Confusion Matrix, Performance Metrics such as accuracy, precision, recall, F1 score and Statistical Tests such as kappa statistics, AUC of ROC curve, which can quantify and pinpoint the performance of each of the models. A confusion matrix can visually show the performance of each of the model by depicting how many instances are correctly classified and misclassified. Table 3(a) provides the confusion matrix while Table 3(b) explains the performance metrices used in this study.

**Table 3(a):** Confusion matrix.

Metrics	Formula/Description			
Confusion Matrix			**Actual**	
			**High Freq**	**Low Freq**
	**Predicted**	High Freq	*True Positive (TP)*	*False Positive (FP)*
		Low Freq	*False Negative (FN)*	*True Negative (TN)*

**Table 3(b):** Performance metrics.

Metric	Definition	Formulae
Accuracy	Accuracy is the proportion of predictions that are correct. It is calculated by dividing the number of correct predictions by the total number of predictions.	$\frac{\text{TP}+\text{TN}}{\text{TP}+\text{TN}+\text{FP}+\text{FN}}*100$
Precision	Precision measures how accurately the model predicts positive instances. It is calculated by dividing the number of true positives by the total number of predicted positives.	$\frac{\text{TP}}{\text{TP}+\text{FP}}*100$
Sensitivity (Recall)	Sensitivity measures the proportion of positive instances that are correctly identified. It is calculated by dividing the number of true positives by the total number of actual positives.	$\frac{\text{TP}}{\text{TP}+\text{FN}}*100$
Specificity	Specificity measures the proportion of negative instances that are correctly identified. It is calculated by dividing the number of true negatives by the total number of actual negatives.	$\frac{\text{TN}}{\text{TN}+\text{FP}}$
F1-Score	The F1 score is a weighted average of precision and recall. It is calculated by harmonic mean of precision and recall. The F1 score is a good metric to use for classification problems where both precision and recall are important.	$2*\frac{\left(\text{Precision}\;*\;\text{Recall}\right)}{\text{Precision}+\text{Recall}}$
Kappa Statistics	Kappa statistics is a measure of how well a model performs compared to a random model. It is calculated by comparing the observed agreement between the model's predictions and the actual labels to the expected agreement under a random model.	$\frac{\text{pa}‐\text{pac}}{\left(1‐\text{pac}\right)}$ where, pa represents total agreement probability and pac represents probability by chance
ROC Curve	The ROC curve is a graphical representation of the trade-off between sensitivity and specificity. It plots the true positive rate (TPR) against the false positive rate (FPR). The higher the AUC (area under the curve), the better the model's performance.	ROC is plotted between Sensitivity and (1-Specificity). The area under the curve (AUC) measures the degree to which the curve is up in the northwest corner.

## 5 Results and analysis

In order to devise a policy for creating sustainable LPG distribution model, the segmentation of customers is akin to identification of customers who are not going to uplift subsequent LPG refills. The prime objective of the presented models in this study is to segment customers (LPG) based on their refill frequency into either a high-frequency (HF) or low-frequency (LF) customer segment.This section presents a comparison of the prediction performance of different machine learning algorithms on different data split and 10-fold cross validation.

### 5.1 Experimental setup and model comparison

State of the art ML models such as Naïve Bayes, Decision Tree, Random Forest, and SVM have been incorporated for making a prediction model of customer's liquefied petroleum gas (LPG) refill frequency. In addition, this study proposes a model Improved Stacked SVM (ISS) and compares its performance with the other model undertaken in this study. In addition to the development and comparison of these models, the feature selection has been explained using explainable AI method where ISS model has been explained with in-depth features and model interaction. The experimental setup of this research has been made for different training-testing split i.e., 50-50 %; 66-34 %; 80–20; and 10-folds cross validations. After testing, a confusion matrix of each classifier is developed from which accuracy, sensitivity, specificity, ROC, and AUC values are obtained for analysis. In addition, some of the statistical techniques such as Kappa statistics is involved to assess the credibility of the proposed ISS model with other state of art models to predict LPG refill frequency.

The performance accuracy of all the models for different splits is depicted in Table 4, where the proposed ISS model shows the better results with accuracy in all experimental setups i.e., 79.56 % for 50-50 training-testing split, 80.56 % for 66-34 split, 80.95 % for 80-20 split and 77.47 % for 10-fold. Random Forest (RF) classifier turned out to be the second-best classifiers in performance accuracy.

**Table 4:** Result of performance accuracy of the classification models.

	Random Forest	SVM	Naïve bayes	C5.0	ISS
50%–50 %	77.84	74.47	66.56	76.52	79.56
66%–34 %	80.83	75.92	67.47	78.41	80.56
80%–20 %	79.58	75.92	68.41	79.12	80.95
10-Fold	76.90	73.71	67.10	76.06	77.47

Table 5 demonstrates the Sensitivity of all the models for different split methods. Sensitivity is a performance metric that measures the proportion of actual positive instances that were correctly predicted as positive by the model which is in this research context is High Frequency (HF) of LPG refill.

**Table 5:** Result of sensitivity of the classification models.

	Random Forest	SVM	Naïve bayes	C5.0	ISS
50%–50 %	67.33	57.43	83.25	63.22	61.43
66%–34 %	67.57	56.23	92.65	75.08	69.01
80%–20 %	69.86	57.26	11.50	70.68	67.40
10-Fold	70.37	53.56	15.70	62.83	66.18

The proposed ISS model in this case too provides satisfactory results together with Random Forest Classifier. The sensitivity of the proposed model is 61.43 % for 50-50 split, 69.01 % for 66-34 split, 67.40 % for 80-20 split and 66.18 % for 10-fold. For sensitivity Random Forest, Decision Tree and proposed model shows promising results.

The specificity of all the models has been demonstrated in Table 6. Specificity is a performance metric that measures the proportion of actual negative instances that were correctly predicted as negative by the model which is in our case is Low Frequency (LF) refill of LPG and is an utmost concern of this research. The proposed ISS model turned out best among other classifiers for predicting negative instances. The specificity of the proposed ISS model is 89.22 % for 50-50 split, 86.43 % for 66-34 split, 87.76 % for 80-20 split, 83.43 % for 10-fold. Naïve Bayes model here exhibits the best performance here but before it can be adjudged the best model we need to perform test for Kappa statistics test compared to other studied models. Here we also observe that Proposed classifier ISS and Random Forest model exhibit comparable specificity.

**Table 6:** Result of specificity of the classification models.

	Random Forest	SVM	Naïve bayes	C5.0	ISS
50%–50 %	83.44	83.55	97.58	83.60	89.22
66%–34 %	87.57	86.03	97.07	80.10	86.43
80%–20 %	84.46	85.28	96.97	83.36	87.76
10-Fold	80.42	83.56	94.75	83.12	83.54

The Kappa statistic (also known as Cohen's Kappa) is a measure of inter-rater agreement or inter-annotator agreement for categorical items. It is particularly useful when assessing the agreement between two or more raters or annotators who categorize items into discrete classes. Kappa statistics considers the possibility of agreement occurring by chance and provides a more robust measure of agreement than simple percentage agreement. The proposed ISS classifier exhibits more robustness in terms of Kappa Statistics. The kappa value is considered as a poor agreement if its value is less than 20 %, fair agreement for 20 %–40 %, moderate agreement from 40 % to 60 %, good agreement from 60 % to 80 %, and excellent agreement for 80 %–100 %.

Kappa statistic of machine learning classifiers studied in this research is shown in Table 7. The results show naive Bayes as a poor model with kappa value less than 10 % for all the experimental set ups. Other models show moderate agreement and can be considered further for building prediction models.

**Table 7:** Result of kappa statistics of the classification models.

	Random Forest	SVM	Naïve bayes	C5.0	ISS
50%–50 %	50.96	42.13	07.42	47.5	52.92
66%–34 %	56.25	44.02	08.01	53.31	56.04
80%–20 %	54.21	44.01	10.67	53.53	56.31
10-Fold	80.42	83.56	04.70	83.12	83.54

**Fig. 4:**
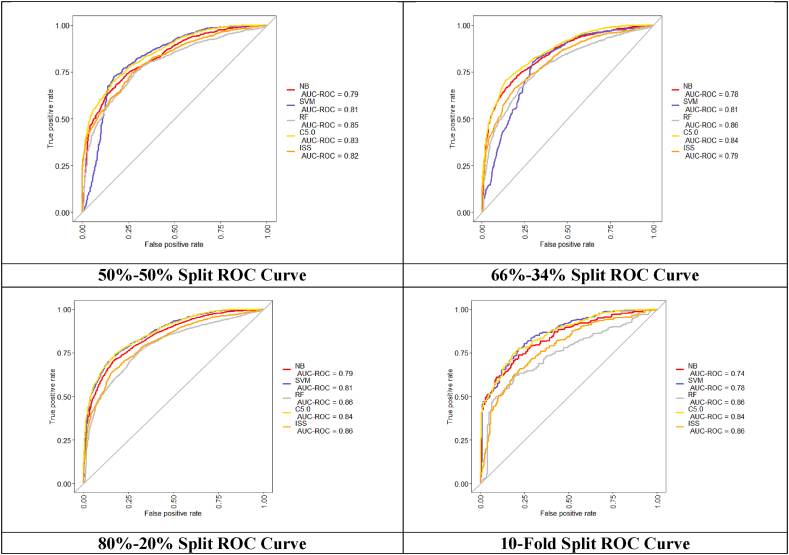
Roc for ML models for different split and 10-fold.

Fig. 4 demonstrates ROC curve (Receiver Operating Characteristic Curve) which is the graph showing the training performance of classification models with respect to different classification thresholds and plots a graph between True Positive Rate (TPR) and False Positive Rate (FPR) and AUC (Area under the ROC Curve) provides an aggregate measure of performance across all classification thresholds. The model which has a higher area under curve value performs better than other models under comparison. With respect to AUC, all the models exhibit an AUC score of more than 75 % and hence the training capability of all the models is good in all experimental setups.

### 5.2 Analysis of explainable ISS model

**Fig. 5:**
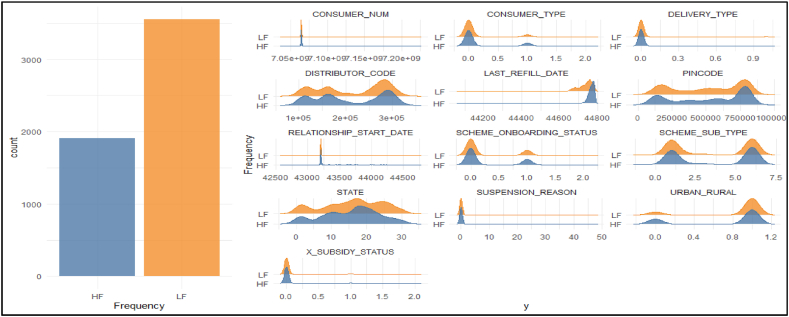
Features information and interaction with target variables.

Going beyond what is apparent from statistical metrics, this study employs Explainable Artificial Intelligence (XAI) to explain the ISS model. All the analysis for explainable ISS has been done for the models trained with 10-fold cross validation. Fig. 5 depicts feature information and their interaction with the different classes (High frequency and Low frequency of LPG refill) of the target variable. It is clear from the figure that both classes are imbalanced (i.e., away from 50 % mark) and is observed that the consumers with high frequency refill are less than consumers with low frequency refill. The same can also be seen in the differences of sensitivity and specificity of the model. It implies that if the classes are balanced, sensitivity of the models may be improved. In addition, the variances of the input features are also explained with respect to both classes of target variable.

**Fig. 6:**
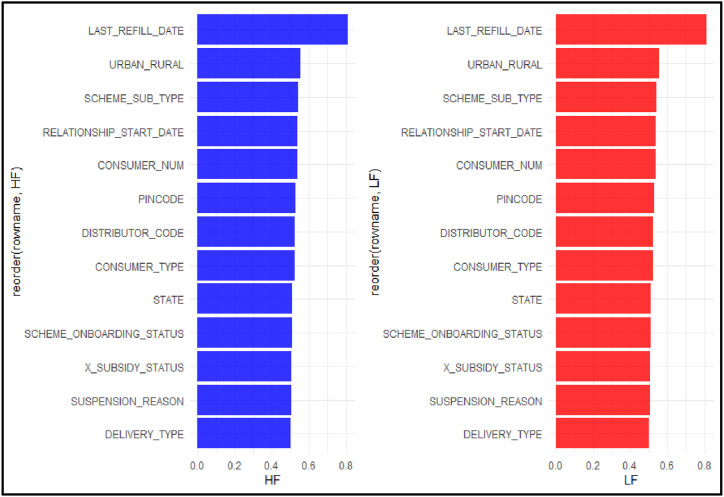
Feature importance.

Features’ importance with respect to ISS model has been shown in Fig. 6. The computed score for each feature of the model simply describes the “importance” of each feature. A high value of feature signifies that the feature has a greater impact on prediction of the instance through the ISS model. It can be inferred from the figure that Last Refill Value plays a significant role in predicting of both classes and is the most significant among the other features. Other features like Urban-Rural, Scheme types etc. are also important.

For assessing the performance of the model and features, two random instances from the test data were taken. The proposed model trained using 10-fold setting was used to predict both test instances (one for High-Frequency and other for Low-Frequency) for in-depth explanation of the ISS model. The combined threshold value is the deciding factor. Based on the combined threshold value, the machine learning algorithm will predict the input data belongs to a particular segment.

**Fig. 7:**
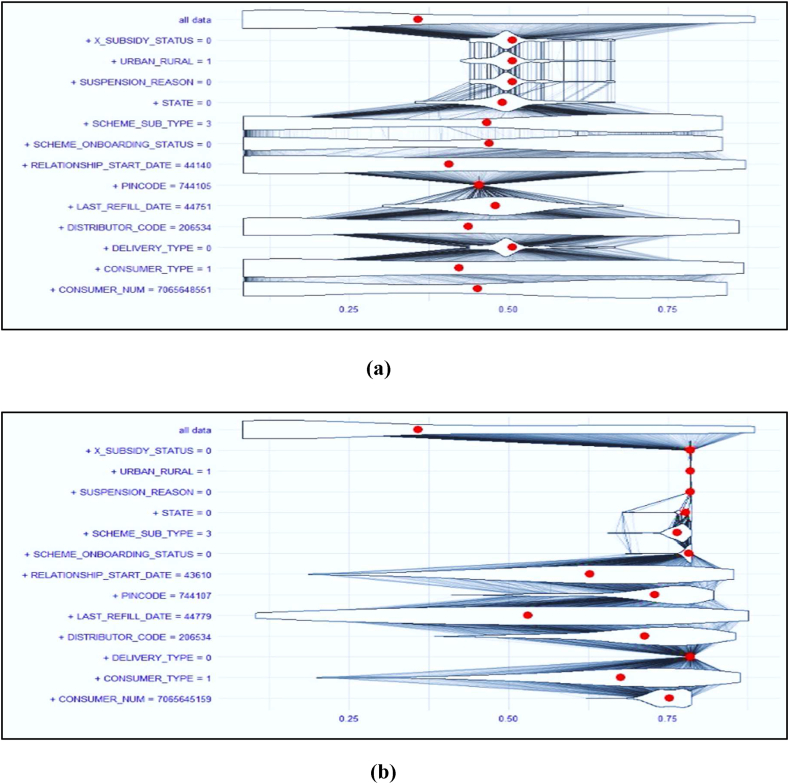
A: Features Contribution in Prediction for high-frequency LPG booking class. B: Features Contribution in Prediction for low-frequency LPG booking class.

Fig. 7(a) is an example of the high-frequency LPG booking class, the threshold value of which is 0.45, which is based on the individual feature value. In figure the red dot indicates each feature's probability value for specific class. The figure depicts the threshold value wherein if threshold greater than 50 % is observed, then there is the high chance that input parameter belongs to the low-frequency segment and if the threshold is less than 50 %, it indicates that the input parameter belongs to the high-frequency LPG segment.

It is observed from Fig. 7(a) that all the feature probability values are equal to or less than 0.5 and the overall combined probability is 0.45, meaning thereby that this input parameter belongs to the high-frequency LPG booking segment.

Similarly, Fig. 7(b), is an example of a low-frequency booking class. All input features shown in Fig. 7(b) have around 70 % probability for a low-frequency class and the combined probability value is 0.78, which is greater than 0.5. This implies that the input parameter under consideration belongs to the low-frequency LPG booking class. In addition, we can observe that the individual feature interaction and specific threshold value for each feature indicates the strength of the feature for consideration into a particular class.

## 6 Discussion

CAG in its report had pointed that even though the PMUY scheme has been rolled out to large population of the targeted beneficiaries, the sustainability of the PMUY was still a challenge [50]. Identification and segmentation of customers’ LPG refill frequency into high and low segments is a prerequisite for design and development of necessary policy interventions to increase the further adoption and ensuring sustainable offtake of LPG refills by the targeted PMUY customers.

Machine Learning (ML) classification models can be used to detect classification thresholds and automatically classify customers into high frequency and low frequency customers. When framing policies based on data and complex algorithmic procedures to classify customers into desired high and low frequency segments, the ML system may use logic which constantly keeps changing and getting transformed. In the process of encoding into a software, ML systems are expected to comply with specific rules and logics. Reasonability, relevance, and fairness are obscured in the process when multiple data sets are being used. A mechanism is thus required that would de-mystify the process of decision making by ML models to users. Whereas ML models have now become mainstream applications being deployed in all imaginable situations, explainable Machine Learning attempts to take a further leap towards making the ML models more understandable.

This study proposes an explainable combined classifier (namely XISS) by stacking random forest (RF) and support vector machine (SVM) classifiers. The ISS classifier is used to classify PMUY customers into low-frequency and high-frequency customers. Based on accuracy, sensitivity and specificity, the Random Forest and ISS classifiers fare better than the other models. The ROC AUC value of 0.846 for Random Forest and ISS models is also the highest. But in terms of Cohens’ kappa value, the ISS model exhibits a higher value when compared to the random forest. On an overall basis, for this dataset ISS algorithm shows slightly higher performance than the random forest.

Also, this research employed Explainable AI to determine and pictorially present the importance of each feature used by the ML model for classifying customers into high and low frequency customers. The probability value for each class for every parameter was calculated and compared with the probability value of each feature to decide to which class it belonged. The overall combined probability for high frequency customer class was 0.45 and for low frequency was 0.78. It is evident from the figures above that for our ISS algorithm, the parameter Last Refill Date plays an especially significant role in decision-making. The other two most significant parameters are Urban Rural customer type and Scheme Sub Type. Thus, the current work employs Explainable AI to get insight into the decision-making process of the ML model.

## 7 Implications of study

### 7.1 Practical implications

Unlike the earlier studies by (Kalli et al., 2022 Gill; Wiehl et al., 2021, 2022) and others which have used stratified sampling to arrive at the sample for study and used statistical significance tests to arrive at feature importance and selection, this study employs a novel approach. In the context of PMUY customers with respect to classification of customers into low-frequency and high-frequency customers, this study employs the Improved Stacked Support Vector Model (ISS) Model which is a hybridisation of Random Forest and Support Vector Machine. This study establishes the superiority of ISS model for classification of PMUY customers LPG refill frequency into High and Low, compared to other state-of-art ML models namely the Random Forest, SVM, Naïve Bayes and C5.0. It employs Explainable Machine Learning (XML) techniques to explain the significance of features used for model development based on the features/dimensions of the provided dataset.

This machine learning approach is appropriate for Big-data scenarios where data is generated at scale and repeated human intervention for addressing model development and arresting model drift is next to impossible.

Machine Learning models being probabilistic in nature, there is a fair chance that deserving customers may be classified as otherwise and would be therefore left out in the targeted schemes owing to the misclassification. Policy implementors need to keep this in mind and build/provision for a separate mechanism/process to bring back the erroneously left out or misclassified customers in this case into the correct segment to ensure the benefits of the schemes flows to the intended beneficiaries.

### 7.2 Managerial implications

Using the ISS method, desired segmentation of customers can be conducted for detection of PMUY subsidy leakages, identification of areas not adequately covered by LPG Distributors, identification of customers who in-spite of financial assistance are not keen to switch to LPG. The classification technique can be extended to classification of distributors into High selling and Low selling distributors based on the no. of customers being serviced and the nos. of refills being supplied. This prediction can form the basis of demand planning for the LPG supply chain and identification of hot spots with respect to LPG cylinder refill shortages. One of the reasons for the low adoption of PMUY scheme in rural areas has been that the LPG Distributors were far off from the residences of the beneficiaries of PMUY scheme. Mappings of regions/districts in terms of High usage and low usage can be helpful in planning a flexible delivery strategy of LPG cylinders. Instead of the customers coming to the Distributors, it is the Distributors who can plan a scheme when to reach the customers. The gauging of performance of Distributors based on the customer satisfaction can also be conducted using the classification engine.

In addition to the intended purpose of the PMUY scheme, GOI through the ecosystem of PMUY Scheme, Direct Benefit Transfer (DBTL) Platform and the JAN Dhan Yojana has created a roust framework through which it can reach out to a large set of the needy population in times of crisis. During the Covid-19 pandemic, GOI had leveraged this framework to give 3 LPG cylinders free of cost to each PMUY beneficiaries. This implies that the technique of classification of PMUY customers with the help XAI can be further used for fine grained customer segmentation and leveraged for directed service delivery by GOI/other interested parties. Also, classification of customers into high and low frequency will allow for identification of customers with high probability of churn out and accordingly either target them specifically with added incentives or exclude them from subsequent benefits as may be deemed necessary.

## 8 Conclusion and Future work

This study started with the aim of developing an Explainable Machine Learning (XML) model to predict the customers refill frequency from Ujjwala scheme so that necessary steps for ensuring sustainable offtake of LPG refills can be taken. The study proposed three research questions which successfully solved by developing Explainable Improved Stacked SVM (XISS) model which gave prediction accuracy up to 81 %. To assure the prediction decisions, proposed model and input features interaction was explained using Explainable AI (XAI) methods. The proposed model compared its result with various state-of-art models through various prediction evaluation metrics and found to be best model in current application scenario. Machine Learning models are considered black box models and are opaque to decision makers. Using Explainable Machine Learning (XML) we were able to get better insights into the working of Machine Learning models with respect to assigning feature importance and feature selection. This model also showed the informative features that participated in prediction using XML. Some of most informative features were Last Refill, Urban Rural customer type and Scheme Sub Type etc. The other features were also ranked with respect to their importance. This insight thereby leads to trust in ML models and the impact of bias in models’ behaviour can be reduced.

The current research can be further extended by using other Machine learning and Deep Learning models for building prediction model in current application domain. Hybridization of the Deep Learning ANN networks with other ML models can be explored as an extension to this study. A contrarian study on the reasons for high adoption of PMUY using same or extended feature sets can throw interesting insights. Further study with respect to drop out rate after initial connection along with its associated LPG refill uptake pattern and the like can also throw interesting insights into the reasons for low intention of LPG refill uptake. Additional information with respect to respect to geographical proximity of LPG Distributors in the area, the service ratings/feedback, presence of competitors, can also be correlated and studied. The proposed model can also be explored in other application domains.

## Data availability statement

Data will be made available on request.

## CRediT authorship contribution statement

**Shrawan Kumar Trivedi:** Project administration, Data curation, Conceptualization. **Abhijit Deb Roy:** Writing – original draft, Visualization, Validation, Software, Resources, Methodology, Investigation, Formal analysis. **Praveen Kumar:** Writing – original draft, Methodology, Investigation, Formal analysis. **Debashish Jena:** Writing – original draft, Software, Resources, Formal analysis. **Avik Sinha:** Writing – review & editing, Supervision, Project administration, Funding acquisition.

## Declaration of competing interest

The authors declare that they have no known competing financial interests or personal relationships that could have appeared to influence the work reported in this paper.
